# Genephony: a knowledge management tool for genome-wide research

**DOI:** 10.1186/1471-2105-10-278

**Published:** 2009-09-03

**Authors:** Angelo Nuzzo, Alberto Riva

**Affiliations:** 1 Centre for Tissue Engineering, University of Pavia, via Ferrata 1, I-27100, Pavia, Italy; 2 Department of Molecular Genetics and Microbiology, University of Florida, Gainesville, Florida 32610, USA; 3 University of Florida Genetics Institute, University of Florida, Gainesville, Florida 32610, USA

## Abstract

**Background:**

One of the consequences of the rapid and widespread adoption of high-throughput experimental technologies is an exponential increase of the amount of data produced by genome-wide experiments. Researchers increasingly need to handle very large volumes of heterogeneous data, including both the data generated by their own experiments and the data retrieved from publicly available repositories of genomic knowledge. Integration, exploration, manipulation and interpretation of data and information therefore need to become as automated as possible, since their scale and breadth are, in general, beyond the limits of what individual researchers and the basic data management tools in normal use can handle. This paper describes Genephony, a tool we are developing to address these challenges.

**Results:**

We describe how Genephony can be used to manage large datesets of genomic information, integrating them with existing knowledge repositories. We illustrate its functionalities with an example of a complex annotation task, in which a set of SNPs coming from a genotyping experiment is annotated with genes known to be associated to a phenotype of interest. We show how, thanks to the modular architecture of Genephony and its user-friendly interface, this task can be performed in a few simple steps.

**Conclusion:**

Genephony is an online tool for the manipulation of large datasets of genomic information. It can be used as a browser for genomic data, as a high-throughput annotation tool, and as a knowledge discovery tool. It is designed to be easy to use, flexible and extensible. Its knowledge management engine provides fine-grained control over individual data elements, as well as efficient operations on large datasets.

## Background

Modern biomedical research is an increasingly *knowledge-intensive *endeavor. New experimental technologies and high-throughput analysis methods produce vast quantities of data with each experiment. Systems biology approaches investigate biological processes on a large scale, relying on the measurement and analysis of thousands of variables in order to elucidate the structure and behavior of complex biological systems. Online databases store an exponentially increasing amount of information, from raw DNA sequences to high-level observations on genotype/phenotype correlations. The shift from *hypothesis-based *to *hypothesis-free *research that is made possible by these technological and methodological advances opens up unprecedented new opportunities for studying biological systems on a large scale, at a low cost, and with a holistic perspective that promises to expand our understanding of biological processes and of their connections with clinically relevant outcomes.

In order to take advantage of this paradigm-changing evolution, researchers will increasingly need effective, practical tools to handle very large volumes of heterogeneous data, both generated by their own experiments and retrieved from publicly available repositories of genomic knowledge [1]. Integration, exploration, manipulation and interpretation of such data therefore need to become as automated as possible, since the "traditional" data inspection and analysis methods are quickly becoming inadequate in a scenario in which an investigator can sample hundreds of thousands of variables in parallel, and an entire new genome can be sequenced and annotated in a matter of days.

While a large amount of work is under way to develop *ad-hoc *analysis methods, able to address the well-known problems related with the statistical significance of results based on a very large number of observations, it is apparent that all phases of the scientific discovery process (hypothesis generation and testing, background knowledge gathering, experiment design, interpretation of results, generation of new knowledge) will have to be adapted to this new reality. The post-genomic era will increasingly require methods and tools able to automatically link new observations and findings to preexisting knowledge.

Finally, new data storage and retrieval systems will need to be developed and adopted in order to handle the unprecedented volumes of data and information being generated in an efficient and productive way. Knowledge and data are represented using nomenclatures, classification schemes and annotation formats that are constantly evolving and often incompatible with each other. Creating, storing and manipulating datasets consisting of hundreds of thousands of records, integrating knowledge from multiple heterogeneous sources, combining and mining data in novel ways for exploratory research, are all tasks that can represent a significant bottleneck for an average researcher who is not an expert in database usage or programming [2].

The ability to effectively address the challenges outlined above will have a direct, dramatic impact on the speed, accuracy and effectiveness of scientific progress in all areas of the life sciences. We are therefore working on developing tools to facilitate the discovery process in high-throughput biomedical research, by providing high usability and effective automation of complex tasks through an easily accessible and intuitive interface. This paper describes Genephony, a powerful online tool designed to assist the non-technical user in creating and manipulating large datasets of genomic information.

## Implementation

Genephony is a Web-based application whose main purpose is to allow the user to easily build *sets *of biological objects. Sets can be created by providing identifiers or query terms, or can be derived from other sets through appropriate transformations. The system automatically keeps track of the relationships among sets, and allows the user to freely navigate through them via a simple, consistent and intuitive user interface. Genephony is designed to be highly interoperable with other online tools: it accepts a wide variety of common formats in input, it provides extensive data export capabilities, and it features a SOAP server interface [3] that allows other software tools to programmatically interact with it.

### The knowledge base

Genephony is able to handle a wide variety of object types, including genomic entities (chromosome regions, genes, transcripts, SNPs, miRNAs, CNVs), classifications and taxonomies (GeneOntology, HomoloGene, pathways), experimental identifiers (probesets for common gene expression and genotyping microarrays), computational predictions (e.g. transcription factor binding sites), and high-level genetic and phenotypic data (e.g. SNP frequencies from HapMap, entries from OMIM and GAD [4]).

The system relies on a local, integrated database of genomic information that includes information about most of the object types mentioned above and, when practical, on real-time access to online resources. It is important to note that Genephony does not try to reproduce exactly the entire contents of all the source databases it uses: doing so would be extremely impractical and ultimately not very useful. Genephony's local knowledge base, instead, represents a selection of the most commonly used object types and data elements, a selection that reflects the needs and requirements of an "average" genomic study. Since the system is based on a modular and general architecture, the default knowledge base described here can easily be replaced with alternative ones that are focused on alternative domains, by defining new object types and new relationships among them.

The choice of maintaining a local database implies an effort to ensure its contents are up to date, through scripts that periodically check the source databases for new data releases. On the other hand, the alternative solution of retrieving the data from the source databases in real time is not practical for a variety of reasons: to start, most online resources enforce a limit on the number and frequency of queries that they accept from a client, making it impossible to work on large volumes of data; not all resources provide interface to access their data in an efficient and machine-friendly way; and finally, accessing very large datasets over the Internet is usually too slow for practical uses.

### Data and object representation

Biological objects are internally represented as data structures composed of several *slots*, each of which contains a single element of information. For example, SNPs (Single Nucleotide Polymorphisms) may be represented by an object containing slots for the SNP identifier (NCBI "rs" number), its genomic location (chromosome and position), its alleles, and its validation status. Each object possesses a unique identifier. Usually this will be the "natural" identifier of the entity being described, when available (e.g., HGNC names for human genes, NCBI "rs" identifiers for SNPs); otherwise one will be internally generated by the system.

A *set *is a collection of objects of the same type. Sets are created by the user by entering query terms, by uploading files, or by performing operations on existing sets. There is no *a priori *limit on the number of objects that a set can contain, or on the number of sets that can be created, and the system is optimized to handle sets containing a very large number of objects. Sets can then be browsed, filtered, annotated and exported in a variety of ways. The next section provides detailed information on all the dataset operations available in Genephony.

## Results

To start working with Genephony, the user creates a session, giving it a unique identifier. No password is currently required, although one may be optionally used to protect data privacy. Once a session has been established, the user can populate it by creating new sets in one of the following ways:

1) Manually entering one or more identifiers. The system is able to automatically recognize a large number of common identifiers; this is accomplished by a set of *autodetect *procedures that examine the supplied identifiers and determine their possible meanings (Table 1 displays a list of the currently recognized identifiers). When multiple identifiers are entered, the system will select the autodetector that applies to the majority of them; although the user has the option of overriding autodetection by manually specifying how to interpret identifiers, this is rarely necessary. After decoding all supplied identifiers, the system creates a set containing the corresponding objects.

**Table 1:** Identifiers automatically recognized by the system

**Object**	**Source**	**Examples**
Genomic regions	UCSC Genome Browser (hg18)	chr3:120,000,000-150,000,000

Cytogenetic bands	UCSC Genome Browser (hg18)	chr3:q13.11, chr3:q13

dbSNP identifiers	dbSNP (build 130)	rs36126692

Entrez GeneID identifiers	NCBI	3456

HGNC gene symbols	NCBI	IFNB1

Genbank mRNA accession numbers	NCBI	NM_002176

SWISSPROT protein identifiers	SWISSPROT	P01574

ENSEMBL gene identifiers	EBI	ENSG00000171855

GeneOntology classes	GO	GO:0051990

OMIM entries	NCBI	MIM:178600, MIM:hypertension

STS markers	UCSC Genome Browser (hg18)	AFM344WE9, GDB:199719

MicroRNA identifiers	Sanger	hsa-mir-942

MicroRNA accession numbers	Sanger	MI0005767

Microarray probesets	Affymetrix, Illumina	208173_at

SNP microarray probesets	Affymetrix	SNP_A-1507458

CNVs	Affymetrix	Variation_0008

GAD entries	GAD	GAD:retinopathy

2) By uploading a file containing identifiers. The system accepts delimited text files and Excel spreadsheets, and handles both ZIP and gzip compression. The user needs only specify the column that contains the identifiers of interest; the identifiers are then parsed and translated into objects using the same procedure described in 1).

3) By deriving them from an existing set, or combining two existing sets. In a *Derive *operation, a new set is generated using the data from a single existing one. For example, given a set of genomic regions, it is possible to generate the set of all SNPs belonging to them. In a *Combine *operation, the data contained in two existing sets is used to generate a new one.

When a set is created, it is initially populated only with the identifiers of the objects it should contain; the object themselves are actually created the first time they are accessed, for efficiency (in some cases a set is only used to derive other sets from it, and the object identifiers may be sufficient for that purpose). The data used to create the object are usually retrieved from the local database through highly optimized queries, although in general they could come from other sources as well (e.g., from real-time access to remote resources).

All generated datasets are permanently stored in the current session. The system keeps track of how each set was generated, and of which other sets were generated by it. It is therefore always possible to reconstruct the path through which any individual dataset was produced, and the user has the option of navigating back to previously-generated sets at any time, in order to examine the data they contain or to generate new sets by following alternative Derive or Combine paths. Moreover, the system records the relationships among individual objects in datasets that are derived from each other. For example, when a set of SNPs is derived from a set of genomic regions as described above, the system will create a table associating each SNP with the genomic region (or regions) it belongs to, and each region with the SNPs it contains. In general, these will be *many-to-many *relationships, and will allow the user to determine how an individual object was produced or how many derived objects were produced by an object in the starting set. These data structures are also used by the *Annotate *command as described below.

### User interface

**Figure 1:**
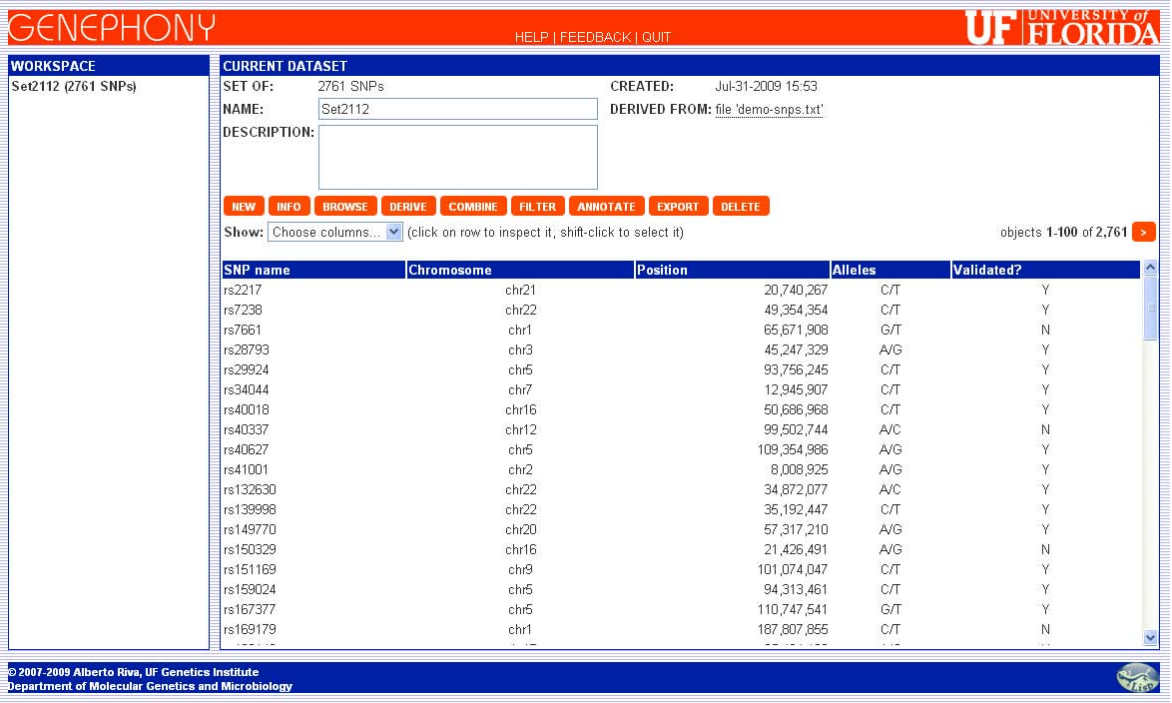
**Genephony main window**. Genephony's user interface is based on a single window divided into three panels. The *Workspace *lists all the sets in the current session. The *Current Dataset *panel displays information about the currently focused set and contains the buttons through which the user can perform all available operations. The third panel displays information about the contents of a set and its relationships with other sets, and is used to receive input from the user. This figure shows the contents of the initial dataset, generated by uploading a text file containing 2,761 SNP identifiers.

Genephony's main interface window, shown in Figure 1, is divided into three panels. On the left is the *Workspace*, which lists all the sets in the current session. The user can "focus" any one of the sets in the session by clicking on its name in the Workspace panel; the currently focused set is shown in bold face. The top right panel displays information about the currently focused set: its name, a description, the number and type of object it contains, how it was generated, and how many other sets it is a parent of (the set's name and description are editable and can be changed by the user at any time). This panel also contains the buttons through which the user can perform all available operations. Finally, the bottom panel is used to display information about the contents of a set, or about its relationships with other sets; it is also used to get input from the user when running certain commands. For example, when the user clicks on the button for the Derive command, the bottom panel will display the list of derive operations available for the current set.

The *New *command opens the initial page and allows for the creation of a new set. The *Info *command displays additional information about the current set that would not fit in the top panel, such as the complete list of sets that were derived from it. The *Derive *and *Combine *commands are used to generate new datasets from the current one as described above.

The *Browse *command displays the contents of the current set as a table in which each row represents an object in the set and each column contains one of the fields of the objects. Several commands are available while browsing: the user may hide or show any column in the table, and sort the set contents by the value of any field by clicking on the header of the corresponding column. Clicking on a table row brings up a page containing detailed information about the object it contains, including the set of "parent" objects that generated it. For instance, considering again the example described above, the page for an individual SNP object would contain the complete list of fields with their values, and the list of genomic regions that contain it (there may be more than one "parent" region for a SNP because genomic regions, in general, may overlap each other). The user may then choose to restrict the display to a manually-selected subset of the rows; the remaining objects in the set are effectively filtered out, as described below.

**Figure 2:**
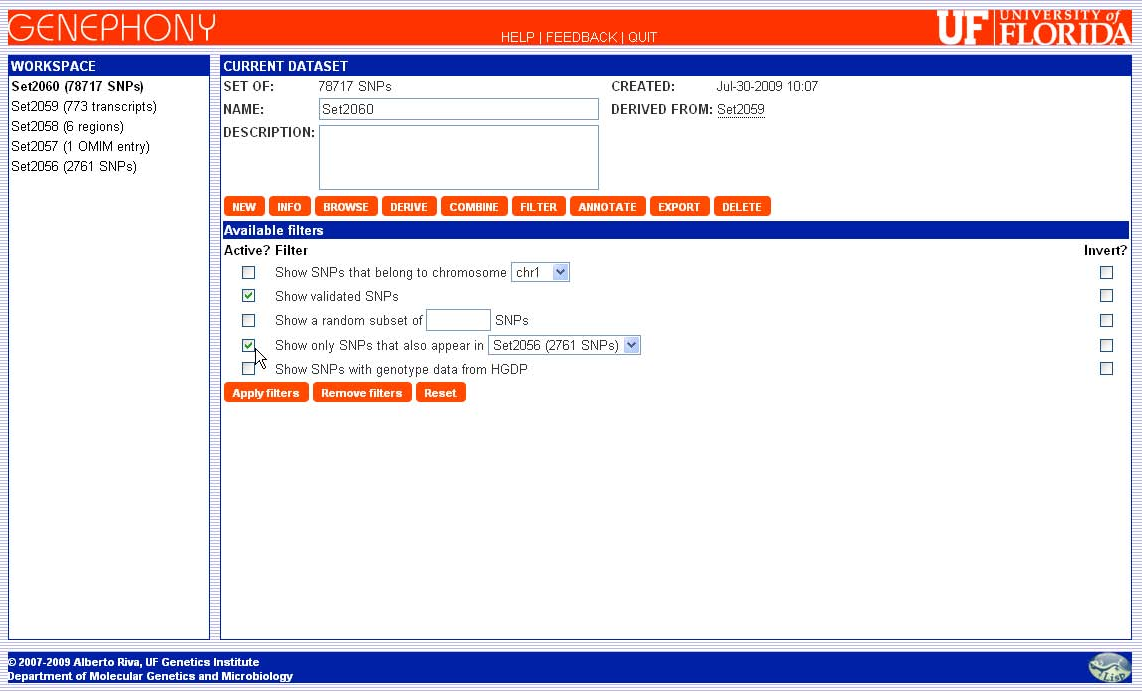
**Dataset filtering**. Datasets can be filtered to display only a subset of the objects they contain. In this example, we apply two filters to a dataset containing 78,717 SNPs: the first one selects *validated *SNPs only, while the second one selects SNPs that also belong to a different set of SNPs. The *Active? *checkboxes are used to indicate which filters to apply, while the *Invert? *checkboxes cause the selected filters to be reversed (ie, only objects that do not satisfy the filter condition will be displayed).

The *Filter *command can be used to hide the objects in a set that do not meet a specified condition. Once a dataset is filtered, all subsequent operations only apply to its visible elements. For example, a set of regions may be filtered to display only the ones belonging to a specified chromosome. If the user then applies the Derive command to derive the set of SNPs they contain, the operation will be applied only to the visible regions, and the resulting set of SNPs will contain only the ones belonging to the regions on the specified chromosome. Filters can be inverted, to select only objects that do not meet the filter condition, and multiple different filters can be applied at the same time, in order to select the objects that meet all specified conditions at the same time (see Figure 2). Finally, filters can be removed bringing the set back to its initial state, with all objects visible.

A very powerful feature offered by Genephony is the *Annotate *command that allows the contents of a dataset to be added to any one of its "parent" or "child" datasets. For example, a set of SNPs can be used to annotate the set of genomic regions it was derived from: the system will keep track of the relationship between each SNP and the region it belongs to, so that when browsing or exporting the set of regions, the system will automatically associate each region with the set of SNPs it contains and display the contents of both datasets side by side (see the example of use of the system in the Results section). It is important to note that this feature works across any number of *Derive *steps, in both directions: a set can be used to annotate its parent, its parent's parent, its child, its child's child and so on. Combined with filtering and with the ability to select individual fields of the objects, the Annotate feature can be used to create richly annotated datasets in a few simple steps.

The *Export *command allows the user to retrieve the contents of a dataset in a variety of different formats, including tab- or comma-delimited text files, Excel spreadsheets, and HTML tables. The files can be directly downloaded or received by email, with optional ZIP or gzip compression, or submitted to the Galaxy online tool [5]. Datasets containing objects that represent chromosome regions can also be automatically uploaded to the UCSC Genome Browser and displayed through its well-known interface. Finally, the corresponding DNA sequences (or their translation into amino acid sequences in any of the six possible frames) can be exported in FastA, Genbank or EMBL format.

### Interoperability

In order to facilitate the exchange of data with other applications, Genephony is designed to accept and to generate datasets encoded in the most common formats, including comma- and tab-delimited text files, Excel spreadsheets, and HTML tables. In addition, Genephony provides a SOAP server interface allowing external programs to use its capabilities independently of the Web interface. The SOAP interface is self-documenting and is fully described in the system's Help pages. Its WSDL definition is also provided to enable the automatic generation of client programs.

### Example of use

In this section we present a detailed example of how Genephony can be used to perform a complex data integration and annotation task. Let us imagine we have performed a genotyping experiment on a large set of SNPs, and that statistical analysis of the results has identified a subset of SNPs that are significantly associated with the presence of a phenotype of interest. In order to better characterize our results, we would now like to determine *which of these SNPs are located in genes that are known to be related to the phenotype*. In the example described here, we used a dataset of 2,761 SNPs, and we chose Insulin-Dependent Diabetes Mellitus (IDDM) as the disease under study (for those readers wishing to walk through this example, the input file containing the SNP identifiers is available in the "Tutorial" section of the program's Help pages, along with step-by-step instructions).

Our strategy will be to query a database of genotype-phenotype correlations, such as OMIM or GAD, for genes contained in regions known to be associated with the disease, to extract the SNPs contained in their transcripts, and to intersect this set of SNPs with the original set. To start, we create a new session and upload the input file using the "Create Dataset" form, specifying that the identifiers are in column 1. The system automatically parses the "rs" identifiers contained in the file and creates an initial set of 2,761 SNPs, that can be displayed using the Browse command (see Figure 1). Next, we turn to the problem of identifying genomic regions associated with diabetes. One possible way to do this is by querying the OMIM database: we return to the "Create Dataset" screen and enter the query term "MIM:diabetes" in the "Enter region or identifier(s)" field (the MIM: prefix is used to indicate that the following term should be interpreted as part of an OMIM entry title). This results in a set containing the 89 OMIM entries containing the word "diabetes" in their title. We then use the Browse command to display the contents of this dataset, locate the row containing OMIM entry 222100, "Diabetes Mellitus, Insulin-Dependent, IDDM", and select it by shift-clicking on it. Clicking on the "click to filter" command appearing at the top of the browse window, we filter the OMIM set restricting it to this single entry. We can now exploit the information on genomic regions associated with phenotypes provided by OMIM to create a set of regions, using the appropriate Derive command; the result in this case consists of the six genomic regions. With a further Derive operation we create the set of all 773 transcripts contained in these regions and, in turn, of the 78,717 SNPs contained in them.

**Figure 3:**
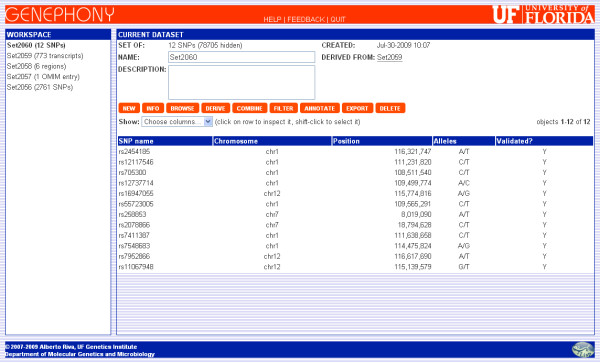
**Result dataset**. The dataset of 12 SNPs resulting from the data integration example described in the text. These SNPs are members of the initial set of 2,761 SNPs generated from the uploaded file, are validated according to dbSNP, and belong to transcripts in regions known to be associated with IDDM (according to the OMIM database).

In order to answer our original question, we just need to find the SNPs that appear both in this set and in the one uploaded at the beginning of this session. This is accomplished using the Filter command, since one of the available filters restricts the set to the SNPs that also appear in another set. We apply this filter together with a second one that only displays validated SNPs, as shown in Figure 2, and the result is the set of 12 SNPs shown in Figure 3.

**Figure 4:**
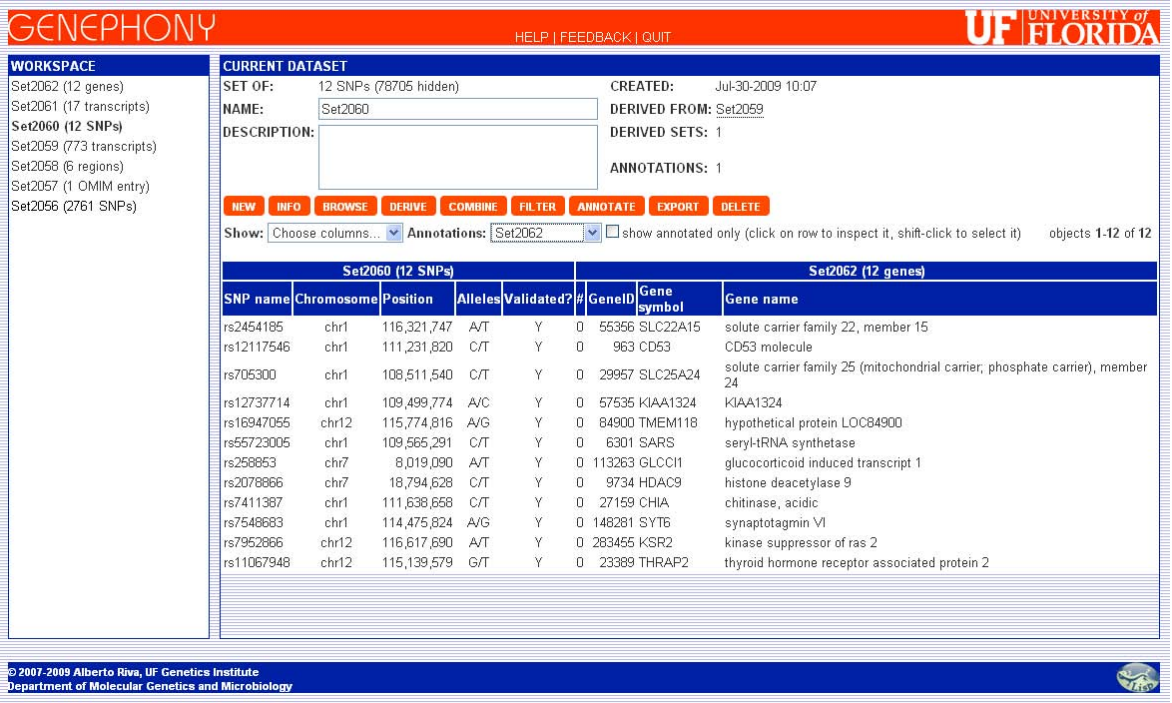
**Annotated results**. Using the *Annotate *command, the final set of SNPs can be annotated with information about the genes the SNPs belong to (found in Set2062, that is derived from the set of SNPs through an intermediate set of transcripts). The contents of the two datasets can thus be displayed side by side and exported as a single table.

To conclude, we would like to annotate the resulting set of SNPs with information about the genes they belong to. We start by creating the set of transcripts containing the SNPs and the set of genes producing these transcripts, using two more Derive operations (it is important to note that the Genephony knowledge base treats genes and transcripts as distinct objects, since the same gene may produce multiple transcripts having a different layout on the chromosome). To simplify the display, we use the "Choose columns" menu to select just the GeneID, Gene symbol, and Gene name columns. Then, using the "Annotate" command we annotate these genes with the set of SNPs they were derived from. To view the resulting annotated dataset, we select the set of 12 SNPs from the workspace window, browse it, and select the set of genes from the "Annotations" menu (see Figure 4).

To summarize, using Genephony we were able to quickly identify a set of SNPs belonging to genes that are known to be involved in IDDM and for which we have genotype data in our dataset, a task that would have otherwise required accessing at least three different databases and performing complex data integration steps on large datasets.

## Conclusion

As the life sciences increasingly become knowledge-intensive disciplines, every effort aimed at facilitating the production, organization and dissemination of new knowledge is bound to have a profound effect on the speed, accuracy and effectiveness of scientific research, and of genome-wide, hypothesis free research in particular. Data and information production in this new era is measured on extraordinarily large scales: just in the field of sequencing, massively parallel DNA sequencing systems have increased our sequencing capacity to hundreds of millions of base-pairs per process run. Microarray technology for gene expression or genotype analysis is undergoing a similar evolution, with modern platforms now reaching one million simultaneous measurements. Parallel advances are taking place in proteomics, transcriptomics and metabolomics. This is having a profound effect on genomics-based research throughout the full range of biological science: whole-genome studies that were once unfeasible are now within the possibilities of any medium-sized laboratory, the distinction between model and non-model organisms has been blurred, and it is now possible to directly sequence entire collections of microbes, and viruses.

Genephony is an online tool aimed at researchers who need an easy, practical way to annotate, integrate and explore genomic knowledge and data resulting from large-scale experiments. The system is robust, efficient and extremely easy to use: it automatically determines which operations are applicable on each dataset, and presents them to the user in a detailed, readable form. Identifiers are automatically recognized and converted in order to establish relationships between different datasets. Interval operations are available for all objects that represent regions on chromosomes (e.g. transcripts, binding sites). Very complex sequences of data manipulations can thus be performed in just a few steps, and no knowledge of the structure of the underlying database is required.

Compared to similar systems such as Galaxy [5], DAVID [6], or BioMart [7], Genephony offers a more explicit and general representation of biomedical object types and of the relationships among them (as opposed to Galaxy's flat-file model or DAVID's gene-set centric view), a flexible workflow model that does not constrain the user on a predefined analysis or annotation path, leaving him/her free to generate and combine datasets in an exploratory way, and powerful data reuse and interoperability features. Moreover, Genephony does not enforce a limit on the size of the datasets the user can use, thus making it possible to operate on the entire contents of a set at once regardless of its dimensions.

Genephony does not currently offer graphical output capabilities, since its main focus is on knowledge and information management, but it provides flexible ways of exporting the contents of its datasets in standard formats for use in external visualization and data manipulation tools such as the UCSC Genome Browser and Galaxy. Although it is not an analysis tool, its rich knowledge base makes it suitable for scenarios ranging from basic genomic data annotation to translational research applications aimed at establishing links between the genomic level and medically relevant phenotypes.

The manipulation and interpretation of very large datasets represents a significant bottleneck for researchers who are not experts in database technology and programming. By providing them with effective tools to perform these increasingly common tasks, Genephony has the potential to accelerate the process of turning experimental data into verifiable hypotheses and biomedically relevant findings. Genephony could also be used as a platform for the dissemination of domain-specific knowledge, since its modular nature facilitates the creation of customized knowledge bases. It can therefore be helpful in making biomedical information available and accessible outside the boundaries of research community, resulting in an added benefit for the general public.

## Availability and requirements

• **Project name**: Genephony

• **Project home page: **

• **Access policy: **the system is freely available to anybody. Users are asked to enter a session identifier to start using the system. Using an e-mail address as the identifier is preferable, but is not required.

• **Operating system(s): **Platform independent

• **Programming language: **Common Lisp, Java

• **Any restrictions to use by non-academics: **freely available.

## Authors' contributions

AR designed the Genephony system and was responsible for its implementation. AN contributed to the development of the SOAP interface and other interoperability features. Both authors read and approved the final manuscript.
